# P-1465. Beyond Income and Education: Determinants of Vaccine Attitudes Among Caregivers in a Low-Resource Setting

**DOI:** 10.1093/ofid/ofaf695.1651

**Published:** 2026-01-11

**Authors:** Laura P Mendez-Reyes, Jeannette Llaneza, Isabel Cintron, Emely P Veloz, Claudio Andujar, Denisse M Román, Filgia L Pimentel, Massiell L Rosario, Nadime Hasbun, Maritza Ramirez, Hector Jose Lora-Rodriguez, Robert Paulino-Ramirez

**Affiliations:** Universidad Iberoamericana, Distrito Nacional, Distrito Nacional, Dominican Republic; Univeridad Iberoamericana, Santo Domingo, Distrito Nacional, Dominican Republic; Hospital Pediátrico Dr. Robert Reid Cabral, Santo Domingo, Distrito Nacional, Dominican Republic; Universidad Iberoamericana, Distrito Nacional, Distrito Nacional, Dominican Republic; Universidad Iberoamericana, Distrito Nacional, Distrito Nacional, Dominican Republic; Universidad Iberoamericana, Distrito Nacional, Distrito Nacional, Dominican Republic; Universidad Iberoamericana, Distrito Nacional, Distrito Nacional, Dominican Republic; Universidad Iberoamericana, Distrito Nacional, Distrito Nacional, Dominican Republic; Universidad Iberoamericana, Distrito Nacional, Distrito Nacional, Dominican Republic; Hospital Infantil Dr. Robert Reid Cabral, Distrito Nacional, Distrito Nacional, Dominican Republic; Yale New Haven Hospital, Distrito Nacional, Distrito Nacional, Dominican Republic; Universidad Iberoamericana, Distrito Nacional, Distrito Nacional, Dominican Republic

## Abstract

**Background:**

Despite public health initiatives, childhood vaccination coverage remains incomplete in the Dominican Republic (DR), with approximately 15% of children not fully immunized according to WHO and UNICEF. Vaccine hesitancy (VH) among caregivers has emerged as a potential barrier to achieving optimal immunization rates. The objective of this study was to elucidate the determinants of VH among parents and caregivers, with a particular focus on identifying socioeconomic and informational factors.

**Methods:**

A cross-sectional survey was conducted among 186 parents and caregivers at a national pediatric referral hospital in the DR. Participants completed digital and printed versions of the Parent Attitudes about Childhood Vaccines (PACV) survey. Additional items were included to collect demographic and socioeconomic data. Descriptive and inferential statistics were performed using JASP software.

**Results:**

Of the 186 respondents, 165 were mothers, with a mean age of 30 years (SD = 8.9). Most families reported having 2-3 children (56.45%), with 63.98% of participants having less than a high school education and 60.22% reporting a monthly household income < 333 USD. Despite these socioeconomic challenges, 96.77% of participants exhibited favorable attitudes (FA) toward childhood vaccination (mean PACV score: 18.38, SD = 14.55). FA were significantly associated with a reduced likelihood of being influenced by vaccine misinformation on social media (χ2 = 3.956, df = 1, p = 0.047) and were more common among not formally employed caregivers (χ2 = 10.165, df = 3, p = 0.017). Monthly household income and educational attainment were not significantly associated with vaccination attitudes (p > 0.05).

**Conclusion:**

Low educational attainment and limited income were prevalent but did not significantly predict vaccine hesitancy. Contrary to initial hypotheses, most of the parents and caregivers demonstrated FA towards vaccination, particularly those not formally employed. These findings suggest that barriers to full immunization coverage in the DR may extend beyond socioeconomic determinants, implicating broader structural, didactic and cultural influences. Targeted interventions addressing misinformation and improving healthcare access are warranted to close existing immunization gaps.

**Disclosures:**

All Authors: No reported disclosures

